# Cerebral Myelination in a Bronchopulmonary Dysplasia Murine Model

**DOI:** 10.3390/children10081321

**Published:** 2023-07-31

**Authors:** Wenwen Chen, Ran Wang, Chao Chen

**Affiliations:** 1 Children’s Hospital of Fudan University, Shanghai 201102, China; pipixiu@163.com (W.C.); 20111240016@fudan.edu.cn (R.W.); 2 Key Laboratory of Neonatal Diseases, National Health Commission, Shanghai 201102, China; 3 Zhangzhou Municipal Hospital of Fujian Province, Zhangzhou 363000, China

**Keywords:** neonatal hyperoxia, molecular injury, cerebral myelination

## Abstract

Introduction: Bronchopulmonary dysplasia (BPD) is a devastating disease in preterm infants concurrent with neurodevelopmental disorders. Chronic hyperoxia exposure might also cause brain injury, but the evidence was insufficient. Methods: Neonatal C57BL/6J mice were exposed to hyperoxia from P0 to induce a BPD disease model. Lung histopathological morphology analyses were performed at P10, P15, and P20. Cerebral myelination was assessed using MBP (myelin basic protein, a major myelin protein), NfH (neurofilament heavy chain, a biomarker of neurofilament heavy chain), and GFAP (glial fibrillary acidic protein, a marker of astrocytes) as biomarkers by western blot and immunofluorescence. Results: Mice exposed to hyperoxia exhibited reduced and enlarged alveoli in lungs. During hyperoxia exposure, MBP declined at P10, but then increased to a comparable level to the air group at P15 and P20. Meanwhile, GFAP elevated significantly at P10, and the elevation sustained to P15 and P20. Conclusion: Neonatal hyperoxia exposure caused an arrest of lung development, as well as an obstacle of myelination process in white matter of the immature brain, with a decline of MBP in the generation period of myelin and persistent astrogliosis.

## 1. Introduction

Neonatal hyperoxia exposure is frequent among preterm infants for lifesaving, particularly among those born with respiratory distress before 32 weeks of gestational age. For infants born preterm, even breathing the room’s normal air is hyperoxia relative to the fetus’ environment. Hyperoxia adds oxidative stress to the process of subsequent organ development after birth that might lead to developmental disturbances. Notably, hyperoxia is considered to be the key contributor to bronchopulmonary dysplasia (BPD). BPD seems to be a predictor of functional, behavioral, and sensory deficits [1]. As shown by the literature, lower cognitive scores assessed by Bayley III were more common in preterm-born children with BPD at 18–24 months of corrected age [2]. However, the mechanism underlying neurodevelopmental disturbances in BPD infants are not completely understood.

Encephalopathy of prematurity (EoP) is a major pattern of brain injury in preterm infants, characterized by widespread hypomyelination or diffuse white matter injury (WMI). It is generally accepted that EoP is caused by systemic perinatal inflammation from infection and/or hypoxia-ischemia, with strong evidence revealed by animal models that mimic perinatal conditions [3,4]. Recently, hyperoxia-induced brain injury has attracted attention, since it represents a realistic clinical insult that preterm infants encounter during the transitional period at birth. Several experimental studies demonstrated that hyperoxia damaged mitochondrial function in the brain [5], induced oligodendrocytes degeneration [6], and generated long-term cognitive deficits [7]. During normal human brain development, the formation of myelin sheaths by oligodendrocytes spurts a rapid brain growth that is known as cerebral myelination, at around 30 weeks of gestational age until two years of age, whereas the growth spurt of myelin in rodents is around postnatal day 2 (P2) to P10 [8]. Thus, infants born during this key period are vulnerable to brain injuries. Whether BPD-associated brain injury is caused by intermittent or continuous hypoxia owing to deteriorative lung function or attributed to direct hyperoxia injury is controversial. Direct evidence of negative effects that hyperoxia imposes on the process of myelination is still lacking.

In view of this, we addressed the impact of neonatal hyperoxia on the process of myelination in the same experimental model as BPD, with the aim to reveal the molecular substratum of hyperoxia-associated brain injury from a dynamic perspective.

## 2. Methods

### 2.1. Animals and Hyperoxia Intervention

C57BL/6 J mice were purchased from JieSiJie Laboratory Animal Co., Ltd., Shanghai, China, and were housed in animal care facilities at Fudan University Affiliated Pudong Medical Center, Shanghai, China (approval code: XYXKHU2020-0005). Animal procedures were performed in accordance with the Institutional Animal Care and Use Committee (IACUC) of Fudan University (approval code: 00033).

The female adult mice mated 3:1 with the male in the afternoon and were separated the next morning. To establish hyperoxia-induced models, newborn pups were exposed to 80% hyperoxia at birth (postnatal day 0, P0) along with their mothers, in cages in an airtight Plexiglass chamber (size 80 cm × 35 cm × 30 cm with two holes on the side walls for inputting oxygen and air escape) with a continuous import of oxygen (1 L/min). The fraction of inspiration oxygen was measured by an oxygen analyzer to maintain 80% in the hyperoxia group. This method and oxygen concentration were adopted generally by researchers to stably establish an experimental BPD phenotype [9]. Animals were raised at 22~27 °C with 50~70% humidity and subjected to a 12 h light–dark cycle. Soda lime was used to absorb CO_2_, and silica gel beads were used to absorb H_2_O. Hyperoxia exposure lasted 10 days, 15 days, and 20 days, respectively, and pups were sacrificed at corresponding time points at P10, P15, and P20 for lung and brain harvest. Age-matched control mice were housed in normal room air. Pups were kept with their mothers from the start to the end in the hyperoxia environment, and mothers were exchanged between air and hyperoxia groups every 24 h to avoid oxygen toxicity. Mice were grouped by block randomization. At the time of tissue harvest, mice were euthanized by 5% isoflurane inhalation. The chest cavity was exposed, the left auricle was cut, and the lungs were cleared of blood by perfusion with cold PBS via the right ventricle. The lungs were inflated with 4% paraformaldehyde under constant pressure of 30 cm water until the edges swelled and allowed to fix in 4% paraformaldehyde for 24 h for further paraffin section following dehydration. The brains were removed with careful elimination of the olfactory bulb after a scission at the back of the neck, posterior median line, and skull. A portion of the brain tissues were fixed in 4% paraformaldehyde for further freezing, and the others were stored in a −80 °C refrigerator after quick freezing in liquid nitrogen for further western blot analyses.

### 2.2. Hematoxylin-Eosin (HE) Staining and Lung Morphological Assessment

HE staining is a convenient and effective method to show cellular morphology and lung structure, providing a merit for recognizing some pathological patterns and parameter measurements. It is generally accepted for pathological diagnosis and morphological assessment of BPD phenotype in research. The fixed lungs were embedded with paraffin after gradient dehydration with a series of ethanol and xylene, and then cut into 3.5-μm-thick sections. Three tissue sections of each pup were selected for analysis. The sections were dewaxed, hydrated, and stained with hematoxylin and eosin. After staining, sections were dehydrated through increasing concentrations of ethanol and xylene, and then observed under an optical microscope. Images were taken at 40 multiplying power by an image acquisition system. Then images were observed on computer by K-Viewer software, and lung morphological analysis was performed on ten fields of each section manually. Radial alveolar count (RAC), representing alveologenesis [10], and mean linear intercept (MLI) [11]—representing the average alveolar diameter—were used for pulmonary morphological parameters. RAC counts were performed by magnifying by 4 times, superimposing the primary images. A perpendicular was dropped from the center of a bronchiole to the edge of the acinus (connective tissue septum or pleura), and the number of alveoli cut by this line was counted. MLI (Lm) calculations were done by magnifying by 10 times, superimposing the primary images. A grid was superimposed over each image, and the number of times the alveolar walls intercepted the grid lines was counted. The equation Lm = (N)(L)/m, where N = number of times the transverses were placed on the tissue, L = length of the transverses, and m = the sum of all intercepts, gave Lm.

### 2.3. Immunofluorescence of Cerebral Myelination

Myelin basic protein (MBP) is one of the most abundant proteins in cerebral white matter, and helps to maintain the correct structure of myelin. Neurofilament heavy chain (NfH) is a neurofilament that contributes to the growth and stability of axons. NfH and MBP are considered axon-specific biomarkers [12], which are generally used in combination to identify white matter axons and wrapped myelin. In this study, immunofluorescence staining was performed for MBP and NfH to facilitate the overview of myelination in periventricular white matter, since it is the most studied anatomical structure involved in preterm brain injury. The fixed brain tissues were embedded with OCT after dehydration with a 20 g/L sucrose solution, and then cut into 8-μm-thick sections. The frozen sections were washed with PBS, blocked with 5% donkey serum, and probed with primary antibodies as follows: mouse anti-mouse MBP antibody (808402, BioLegend, San Diego, CA, USA, 1:100) and rabbit anti-mouse NfH antibody (ab207176, Abcam, Cambridge, UK, 1:1000). After incubation at 4 °C overnight for 16 h, the sections were probed with secondary antibodies as follows: donkey anti-rabbit Cy2 (111-225-003, Jackson, 1:500) and donkey anti-mouse Cy3 (715-165-151, Jackson, 1:500), then incubated at room temperature for 1 h. Then, the sections were stained with 4′,6-diamidino-2-phenylindole (DAPI) for 15 min and mounted with AAT Bioquest, followed by observation under laser scanning confocal microscopy. Pictures were taken at 100 times magnification (10 × ocular and 10 × objective). The parameters were set as follows: DAPI: laser line: 405 nm, PMT detector, gain: 696 V; Cy2: laser line 488 nm, PMT detector, gain: 805 V; Cy3: laser line 552 nm, HyD detector, gain 15%; exposure time: 12 s.

### 2.4. Western Blot of MBP and GFAP

As described above, MBP is the most important biomarker of myelin. So, western blot analysis was performed to quantify MBP expression. Glial fibrillary acidic protein (GFAP) is a biomarker for astrocytes [13]. Astrocytes outnumber neurons in human neocortical white matter, and they are vulnerable and responsive to injury. Once encountered with injury, GFAP increases in astroglial cells and processes [14], which is considered a sensitive indicator of brain injury. So, western blot was also performed to quantify GFAP expression. Total protein was extracted using tissue protein extraction reagent (78510, Thermo, Waltham, MA, USA) mixed with a Protease Inhibitor Cocktail (87785, Thermo) under 99:1 proportion. Every 10 mg of brain tissue was added to 100 μL protein extraction reagent, and then ground in a tissue grinder at a rate of 60 Hz for 1 min. The ground mixture was placed on ice for 40 min, and then centrifuged at a refrigerated centrifuge at a rate of 14,000× *g* at 4 °C for 10 min. The supernatant was absorbed into a new Ep tube with a pipette and centrifuged again for 20 min. The final supernatant was transferred into the new Ep tube for analysis. The concentration of protein was confirmed using the bicinchoninic acid assay (BCA). In brief, the standard protein was diluted into 8 different concentrations by diluent to make the standard curve. The samples were diluted 50 times for measurement. The diluted standard protein and samples were added in 96-well plates with a total of 20 μL in each well (3 wells for each standard protein or sample). Exactly 200 μL of BCA working reagent was added to each well and incubated at 37 °C for 30 min. The 96-well plate was put into the Varioskan LUX Multimode Microplate Reader and measured at A562. The standard curve was drawn according to the concentrations of standard protein and their absorbance values. The concentrations of the samples were calculated according to the standard curve. Then, the protein samples were diluted to a uniform concentration by adding protein extraction reagent, and 5 × sodium dodecyl sulfate (SDS) loading buffer of 1/4 volume was added to adjust the final concentration to 5 μg/μL. The mixtures were denatured in a metal bath at 95 °C for 10 min. For western blot, a total of 20 μg of protein (4 μL) was loaded onto 12% SDS-polyacrylamide gels for electrophoresis and transferred onto polyvinylidene difluoride membranes. The membranes were blocked with 5% BSA in Tris-buffered saline-Tween (TBST) for 2 h, and incubated with primary rabbit anti-mouse antibodies: mouse anti-mouse MBP anti-body (808402, BioLegend, San Diego, CA, USA, 1:2000), rabbit anti-mouse GFAP (80788S, CST, Boston, MA, USA, 1:1000), GAPDH (bas 132004, Absin, Shanghai, China, 1:3000), and beta actin antibody (abs132001, Absin, 1:3000) at 4 °C overnight for 16 h. The membranes were then incubated with goat anti-rabbit IgG-HRP (abs20040, Absin, 1:5000) or goat anti-mouse IgG-HRP (abs20039, Absin, 1:5000) for 1 h. Protein blanks were visualized using super signal west femto maximum sensitivity (34096, Thermo) and photographed using a gel imaging system. Beta actin served as the internal reference, and the ratio of the gray value of the target protein to beta actin was used as the relative protein expression. The calculation of the gray value was performed using Image J software (Fiji, developed by NIH and LOCI, Bethesda, MD, USA).

### 2.5. Statistical Analysis

Statistical analysis was conducted using GraphPad Prism software. Descriptive data were presented as means ± SD. Survival rates were compared via a Log-rank test. Continuous variables were compared between two groups via 2-tailed Student’s *t* test, including 6 samples in each group. A *p* value of <0.05 was considered as statistically significant.

## 3. Results

### 3.1. Persistent Alveolar Arrest during Neonatal Hyperoxia Exposure

**Figure 1:**
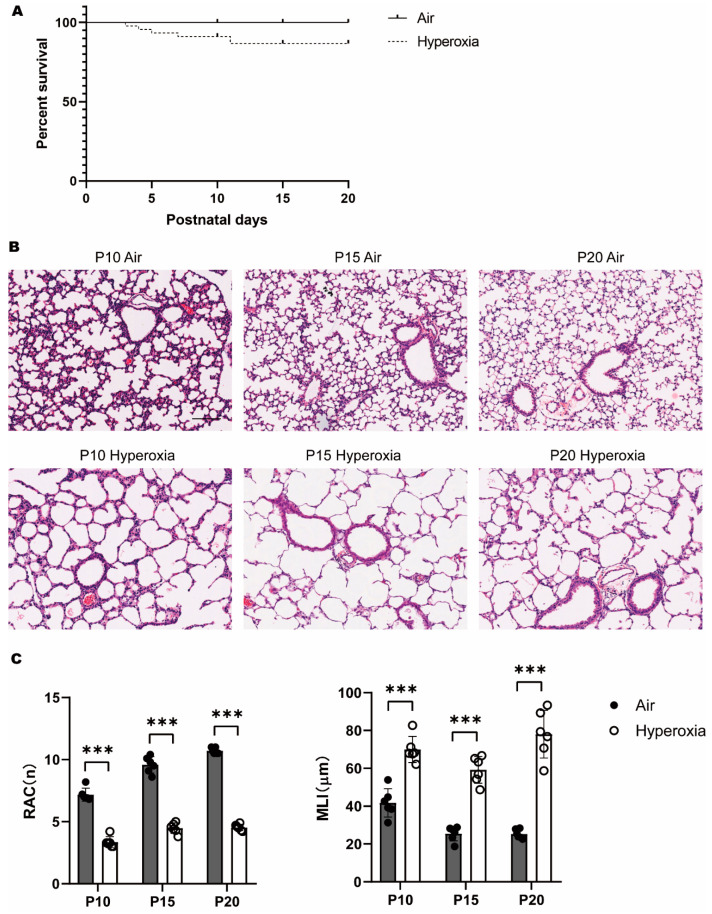
(**A**): Percentage survival of mice after 20 days of room air or 80% O_2_ exposure (*p* = 0.0184). (**B**): Lung morphology at P10, P15, and P20, HE staining. The hyperoxia group showed less alveoli and enlarged alveolar space at P10, and sustained to P20. All the pictures were set on the same scale. Scale bar 100 μm. (**C**): Radial alveolar count (RAC) and mean linear intercept (MLI) counts and statistical graph. The hyperoxia group displayed decreased RAC and increased MLI. Data were calculated of six samples per group and presented as mean ± SD. *** *p* < 0.001.

A total of 6 litters of newborn mice were included in this study. At P0, there were 48 newborn mice without differences in body weight. Hyperoxia exposure decreased the survival rate of pups from P0 to P20 (Figure 1A).

At P10, HE staining showed that in the hyperoxia group, the size of alveoli enlarged, and the number of alveoli and secondary septa declined, while in the air group, secondary septa were abundant, which separated the alveolar sacs, resulting in smaller alveoli and an increased number of terminal alveoli (Figure 1B). Morphological assessment showed that RAC decreased, while MLI increased significantly in the hyperoxia group compared to the air group, which further reinforced the changes by quantifiable index (Figure 1C). At P15 and P20, the enlargement of alveoli became more obvious, with a persistent decline of alveoli and secondary septa. This lung morphology was similar to that of an earlier stage of lung development, and could thus be described as alveolar arrest. These results definitely supported that hyperoxia caused developmental disturbances on the immature lung.

### 3.2. Transient Myelination Impairment during Neonatal Hyperoxia Exposure

**Figure 2:**
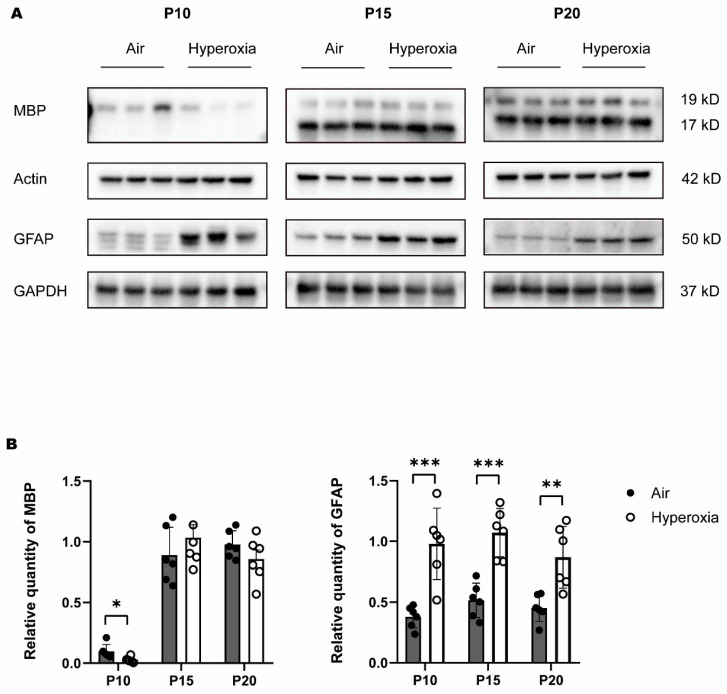
(**A**): Western blot bands of MBP and GFAP in brain tissues at P10, P15, and P20, showing the bands of MBP in the hyperoxia group were lighter than that in the air group at P10, while the bands of GFAP in the hyperoxia group were deeper than that in the air group at P10, P15, and P20. (**B**): Relative quantity of MBP equal to actin, and GFAP equal to GAPDH, showing the difference in MBP between the hyperoxia and air group at P10 was significant, while the differences in GFAP between the two groups at P10, P15, and P20 were significant. Data were calculated of six samples per group and presented as mean ± SD. * *p* < 0.05, ** *p* < 0.01, *** *p* < 0.001.

**Figure 3:**
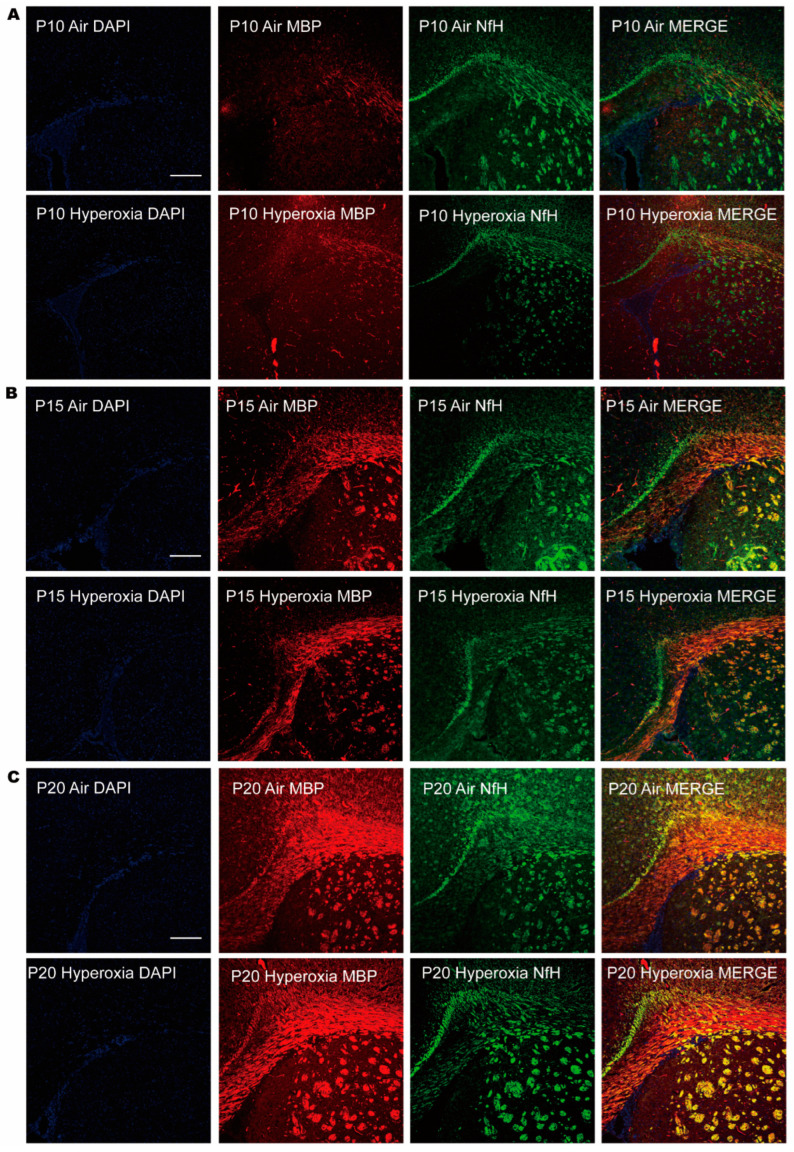
Immunofluorescence staining of MBP (red) and NfH (green) in periventricular white matter at P10 (**A**), P15 (**B**), and P20 (**C**), showing that MBP was inadequate at P10, but became abundant at P15 and P20. During postnatal hyperoxia exposure, MBP declined at P10 in the hyperoxia group, but was restored to the comparable as the air group. All the pictures were set on the same scale. Scale bar 50 μm.

To access the myelination, we examined the expression of MBP (myelin basic protein, a major myelin protein) in the brain tissues through quantitative analysis by western bolt analysis combined with GFAP and qualitative display by immunofluorescence, combined with NfH. Western blot analysis showed that MBP was significantly lower in the hyperoxia group at P10 compared to the air group; at P15 MBP, it was raised to a comparable level as the air group, and at P20 MBP, it did not differ significantly in both hyperoxia and air groups (Figure 2A,B). The results indicated that cerebral myelination in the immature brain was impaired by hyperoxia. To reinforce hyperoxia-induced brain injury, we also examined the expression of GFAP (glial fibrillary acidic protein, a marker of astrocytes in the brain tissues). Western blot showed that GFAP increased significantly in the hyperoxia group at P10 compared to the air group, and the difference sustained to P15 and P20 (Figure 2A,B). The immunofluorescence showed corresponding dyeing of MBP and NfH in the corpus callosum (Figure 3). At P10, neurofibrillary dyed as green and red was thinner in the hyperoxia group than in the air group, and at P15 and P20, the neurofibrillary seemed to be the same thickness in both hyperoxia and air groups. These results provided direct molecular evidence of a negative effect of hyperoxia on the process of myelination. Myelination was frustrated with the decline of MBP and the elevation of GFAP at the early stage of the brain development. Although MBP was restored later, GFAP elevation continued. Cerebral myelination was impaired by hyperoxia at a critical developmental period, and left molecular evidence of persistent damage.

## 4. Discussion

White matter lies beneath the gray matter cortex, which is composed of millions of bundles of axons (nerve fibers) that connect neurons in different brain regions into functional circuits [15]. The white color derives from the electrical insulation, which is called myelin, that enwraps axons. Myelination is the process of these myelin sheaths’ formation. Myelin sheaths act to increase the conduction velocity of electrical impulses and improve brain connectivity [16]. Learning a new skill is associated with altered white matter structure, and the damage of white matter will cause impairments in sensory, motor, and cognitive functions [17]. In humans, myelination starts in mid-to-late gestation, which is equivalent to the perinatal and early postnatal ages in rodents [18]. In other words, the analogous time quantum of active myelination in rodents is around P2 to P10, which is congruent with the time preterm infants are born and survive, beginning from around 24–40 gestational age to 2–3 years after birth. In this time period, myelination is incomplete universally and develops dynamically. This critical time quantum was covered in our study, and the total expression of MBP can represent the overall degree of myelination in the brain. As shown definitely in our study, hyperoxia imposed a negative effect on myelination at P10 in rodents, equivalent to postnatal periods of preterm infants and their early childhood. Before this time period, neurons are established, but very few of the axons in the brain have been already myelinated, so communicational signals could barely transfer through neurons without myelin. The evolving myelination of white matter in this critical period contributes to pronounced improvements in cognitive abilities due to more rapid neural communication and integration of the signals across different brain regions involving well-recognized functions, such as vision [19], sensorimotor [20], memory [21], and language [22]. Infants receiving excessive and prolonged oxygen therapy during this period might experience deficits in myelination, resulting in the loss of approach through which signals contact [23]. The decline of MBP also indicated suppressed oligodendrogenesis, a process in which axon-myelinating oligodendrocytes recruit and generate MBP. In general, myelinating oligodendrocytes are differentiated from oligodendrocyte progenitor cells (OPCs), but they fail to differentiate in hyperoxia-induced brain injury due to degeneration and maturation arrest, thus ultimately resulting in frustrated myelination. As myelin matured, the difference in MBP expression between the hyperoxia and air group became inconspicuous at P15 and P20, which was a slow or quiet time since myelination is almost complete, equivalent to adulthood in humans. It was interesting that the observed micro quantificational changes of MBP did not sustain into adulthood. A speculated reason might be that an acceleration in the developmental trajectory of myelination would have happened in the healing process following hyperoxia injury. There is a potential backup pool of OPCs during adulthood, which are ready for myelin regeneration after injury [24,25]. Furthermore, OPCs are more resistant to oxidant stress in more mature bodies [26], so the decline of MBP caught up. However, MBP is not only the structural protein of myelin, but also an integral driver for myelin compaction via actin disassembly during myelin wrapping [27,28]. Although oligodendrocytes could survive and continue to express myelin genes in response to injury, they fail to maintain compacted myelin sheaths [29]. The compensation of MBP production through an excess of OPCs being generated after injury might not be integrated into neural circuits, thus causing failure in myelin remodeling [30]. Chang JL et al. demonstrated that hyperoxia caused abnormal myelin sheath formation and resulted in impaired myelin integrity, which contributed to WMI [31]. On the other hand, changes in the early developmental period may also play a role in the pathogenesis of EoP, with attention deficit and hyperactivity disorder, anxiety, and autism spectrum disorder as the most prevalent patterns, even absent of visible structural anomalies in the later life [32]. Khanbabaei M et al. also proposed that an altered developmental trajectory rather than structural anomaly could also be an important component of the etiology of EoP, especially those that referred to a milder degree of neurological impairment affecting mostly cognitive function and an increased risk of psychological disorders later in life [33]. It is not uncommon that children with social disorders have insignificant lesions in MRI imaging [34]. As revealed by Allin M et al., there might be a striking pattern of enhanced growth of the corpus callosum in adolescents born very preterm making up for early deficits, and this acceleration represented a delay of the normal maturation process that might be associated with a neuropsychological outcome [35]. Similarly, Morken TS et al. found that the alterations in white matter development caused by perinatal injury were reversible with age, indicating a maturation delay of the white matter [36]. Of note, the short-term prominent deficits of myelination might translate into long-lasting subtle white matter alterations associated with cognitive impairment or psychological disorders, which dominate the phenotypes of EoP in childhood among preterm children.

Astrocytes are predominant non-neuronal cells in the central nerve system (CNS), providing support for neuronal development; the interaction of astrocytes with neural cells synergistically promotes myelination [37]. Astrocytes have long been considered the major inhibitor on CNS repair under harmful stimulus. Previous studies demonstrated that astrocytes could exert potent proinflammatory functions as their primary mode of action after CNS injury [38]. Evidence has shown that neonatal myelination deficits are associated with neuroinflammation that could cause OPCs degeneration brought by astrocytes [39], and this might persist into adulthood [40]. Nowadays, it is recognized that astrocytes also play important roles in CNS repair and remyelination [41]. As a response to injury, the neurotransmitter adenosine 5′-triphosphate (ATP) is released from axons and activates receptors on astrocytes, causing them to release a cytokine that promotes oligodendrocyte development and thus increases myelination. That is, astrocytes can have a priming negative effect on myelination in early injury, as well as participating in repair and recovery after the insult. However, in chronic WMI, the differentiation of offsetting regenerative late ligodendroctye progenitors (preOLs) was hindered in the diffuse astrogliotic lesions, which was known as preOLs maturation arrest with a failure to generate myelin [34]. The reactive astrogliosis usually marked CNS structural lesions, with the elevation of GFAP, which was released after injury as the most important structural protein, labeling extensive branching of astrocytes in white matter [42]. As shown in our study, the decreased MBP expression did not sustain with age and prolonged hyperoxia exposure, but the increased GFAP expression continued, indicating a lasting reactive astrogliosis induced by hyperoxia. The latter might probably result in glial scar formation. In human beings, necrotic lesions (microscopic cysts) in WMI could evolve into glial scars over the course of several weeks, which is a persistent hallmark of brain injury [34].

A dilemma in the explanation of BPD-associated EoP is that BPD infants might encounter intermittent hypoxia frequently due to their immature respiratory control and poor lung function, which could also contribute to WMI. What cannot be ignored is that in spite of continuous oxygen therapy, premature infants experience multiple episodes of hypoxemia [43]. It is well documented that intermittent hypoxia elicits oxidative stress responses that occur during the re-oxygenation period [44]. Several studies demonstrated that recurrent hypoxia or hypoxia-hyperoxia encounters augmented oxidative stresses that generated a robust non-myelination preOLs accumulation relative to lesions in white matter compared to a single episode of hypoxia or hyperoxia [45,46]. In this experimental hyperoxia BPD model, mice could survive without respiratory dysfunction, excluding the effect that might be caused by hypoxia. Previous studies had already demonstrated that mice in this hyperoxia model maintained normal arterial oxygen levels [7]. Hence, the negative impact on the cerebral myelination was confirmed to come from direct hyperoxia injury. This result provided the rationale to avoid excess oxygen in clinical practice. However, extremely preterm infants could seldom survive without oxygen therapy due to immature lung and respiratory control at birth and within early days postnatal, raising challenges for neonatologists guarding against hyperoxia while avoiding hypoxia [47]. Lung-protective ventilation strategies have been proposed as the most important component in the management of preterm infants, such as optimizing gas exchange via volume-targeted ventilation during mechanical ventilation (MV) if necessary, avoiding MV if possible, optimizing noninvasive respiratory support, and setting a SpO_2_ target of 90–95% in monitoring by pulse oximetry [48]. An important concept must be kept in mind—avoiding both prolonged periods of hypoxia (SpO_2_ < 80%) and fluctuations in SpO_2_ [49].

In this study, we demonstrated concomitant brain injury at the molecular level in a BPD murine model. The strength of this study was that the hyperoxia model we applied involved both lung and brain injuries that mimic preterm birth conditions well. The results provided a rational explanation of BPD-associated EoP based on molecular mechanisms. Molecular changes were dynamically inspected, which provided a panoramic understanding of the impact of hyperoxia on the developmental brain.

There were still several limitations in this study. First, whether the lung-brain axis acted in the development of lung and brain injury in this hyperoxia model was not considered. Second, although pathological changes in this hyperoxia animal model were typical, the clinical relevance of the results has to be interpreted with caution because of the differences in conditions where alveolarization and brain development take place between rodents and humans. In addition, this chronic hyperoxia-induced lung injury model may not necessarily mimic BPD [50]. Further research is needed to validate the findings in large animal models of prematurity, which will enable the improvement of clinical translation.

## 5. Conclusions

Neonatal hyperoxia exposure caused an arrest of lung development, as well as an obstacle of myelination process in the white matter of the immature brain, with a decline of MBP in the generation period of myelin and persistent astrogliosis.

## Data Availability

Data presented in this article are available on request from the corresponding author.
